# The Influence of Calcitonin Gene-Related Peptide on Cerebral Hemodynamics in Nonmigraine Subjects with Calcitonin Gene-Related Peptide-Induced Headaches

**DOI:** 10.1155/2021/5540254

**Published:** 2021-04-24

**Authors:** Matija Zupan, Marjan Zaletel, Darja Visočnik, Bojana Žvan

**Affiliations:** Department of Neurology, University Medical Center Ljubljana, Zaloška Cesta 2, 1000 Ljubljana, Slovenia

## Abstract

**Background:**

Calcitonin gene-related peptide (CGRP) is regarded as an important molecule in trigeminovascular sensitization (TVS). CGRP-induced headaches (CGRP-IH) are evoked by intravascular administration of CGRP in nonmigraine and migraine subjects. CGRP might be associated with vasodilatation of the middle cerebral artery (MCA). It is unclear whether CGRP-induced hemodynamic changes relate to CGRP-IH in nonmigraine subjects.

**Methods:**

Twenty healthy subjects participated in our study. Polymodal recording of mean arterial velocity in MCA (vm MCA), end-tidal carbon dioxide partial pressure (Et-CO_2_), mean arterial pressure (MAP), and heart rate (HR) was employed using transcranial Doppler (TCD) sonography. During the experiment, we administered intravenous infusion of CGRP at a rate of 1.5 mcg/min. The vm MCA, Et-CO_2_, HR, and MAP were determined at time points *T*_0_, *T*_1_, *T*_2_, and *T*_3_. We calculated the responses at different time points and combined them into a single response vm MCA_tot_, Et-CO_2tot_, HR_tot_, and MAP_tot_.

**Results:**

We found significant differences along the time points in vm MCA (*p* = <0.001), Et-CO_2_ (*p* = 0.003), MAP (*p* < 0.001), and HR (*p* < 0.001). The relationship between vm MCA_tot_ and Et-CO_2tot_ was significant and positive (*p* = 0.005). The *t*-test showed significant differences between CGRP-IH and non-CGRP-IH subjects in vm MCA_tot_ (*p* = 0.021) but not in Et-CO_2tot_ (*p* = 0.838), MAP_tot_ (*p* = 0.839), and HR_tot_ (*p* = 0.198). Only vm MCA_tot_ showed a significant relationship with CGRP-IH (*p* = 0.028).

**Conclusions:**

Our study provides evidence for vasodilatation of MCA in relation to CGRP-IH due to intravascular CGRP detected by multimodal TCD. In the context of TVS induced by CGRP, MCA vasodilatation seems to represent an epiphenomenon of the underlying TVS.

## 1. Introduction

Trigeminovascular sensitization (TVS) with neurogenic inflammation is supposed to be the underlying pathophysiology of migraine headaches. Calcitonin gene-related peptide (CGRP) is a putatively important molecule in TVS. It is well known that intravascular CGRP provokes CGRP-induced headache (CGRP-IH) and migraine-like attacks in 62% of migraine patients [1]. Intuitively, TVS could relate to CGRP-IH and migraine-like attacks. In addition, CGRP-IH occurs in nonmigraine, healthy volunteers [2]. Therefore, TVS might be operative in nonmigraineurs.

Vasodilatation of cerebral arteries including the middle cerebral artery (MCA) is regarded as an important substrate of TVS. In patients with migraine, a transcranial Doppler (TCD) study showed that intravascular CGRP induced a decrease in mean arterial flow velocity in MCA (vm MCA) suggesting vasodilatation of MCA. CGRP-IH developed in all patients [3]. In nonmigraineurs, vasodilatation of MCA with intravascular CGRP was found using TCD and 133-Xe single photon emission computed tomography. CGRP-IH appeared in 6/10 subjects [4]. Asghar et al. did not find dilatation of MCA utilizing magnetic resonance angiography (MRA) but an increase in CGRP-IH headache score due to intravascularly administered CGRP in healthy subjects [5]. On the other hand, in a TCD study, Lassen et al. found a decrease in end-tidal CO_2_ partial pressure (Et-CO_2_) induced by intravascular CGRP but not with placebo [3], which may be regarded as an indirect evidence of vasodilation. TCD appears to be a more sensitive method for indirectly detecting small changes of MCA diameter compared to MRA because of the exponential relationship between the flow velocity and a vessel's diameter. Therefore, we infer that arterial velocity is a more sensitive surrogate compared to MRA, which measures changes in the arterial diameter only.

According to recent findings, intravascular CGRP is a potent vasodilator of the cerebral arterial tree, which could reflect in arterial velocity lowering due to vasodilatation of the proximal cerebral arteries [6]. Thus, vasodilation in relation to TVS induced by CGRP might appear in nonmigraineurs and could be enhanced in CGRP-IH subjects. In addition, TCD could be a useful method to investigate the hemodynamic consequences of TVS due to CGRP in healthy subjects. The response to CGRP may involve regulatory mechanisms to maintain constant cerebral blood flow (CBF) during intravenous CGRP infusion. Partial CO_2_ pressure could be important for maintaining constant CBF during CGRP stimulation. Nevertheless, the relationship between Et-CO_2_ and vm MCA changes during intravascular CGRP activity remains unclear. The multimodal TCD monitoring enables the following of several hemodynamic parameters on the same time scale, including vm MCA and Et-CO_2_ during CGRP activity [7].

This study is aimed at exploring whether vasodilatation of MCA occurs in nonmigraineurs after intravenous administration of CGRP using polymodal TCD. We predicted that vm MCA associates with Et-CO_2_, and the response of vm MCA to CGRP is pronounced in CGRP-IH subjects.

## 2. Materials and Methods

This study uses the method of Visočnik et al., and the method description partly reproduces their wording [8].

Twenty healthy subjects participated in our study (9 females aged 37.0 ± 2.8 years and 11 males aged 41.8 ± 7.6 years, *p* = 0.66). The inclusion criteria were age more than 18 years, normal somatic and neurological status, and the absence of hemodynamically significant atherosclerotic changes of the carotid and vertebral arteries as evaluated by color-coded duplex sonography. The exclusion criteria were migraine and other primary headache disorders (including family history of migraine), previous cerebrovascular, endocrine, renal or liver diseases, uncontrolled hypertension, daily intake of medication except for contraceptives, pregnancy, and breastfeeding. The participants were free of tobacco, coffee, tea, or any other food or beverages containing caffeine for at least 12 h before the start of the measurements. All participants were given written explanations about the experimental procedure and were informed that they were free to withdraw from the study at any time. They all gave written informed consent to participate in the study. The National Medical Ethics Committee of the Republic of Slovenia approved the study.

Before the onset of the experiment, color-coded duplex sonography of the carotid and vertebral arteries was performed using the standard procedure and blood samples were drawn for complete blood count, potassium, and sodium. The experiments occurred at 9: 00 am in a quiet and dark room with a constant temperature. During the experiment, the participants were resting in the supine position. TCD sonography with 2 MHz ultrasound probe was applied to measure the vm MCA through the left temporal acoustic window. The signals of the artery were defined according to the direction of the blood flow, the typical depth of the signal, and the response to compression. A mechanical probe holder was used to ensure a constant probe position. During the entire experiment, the mean blood pressure (MAP) and heart rate (HR) were continuously measured using noninvasive plethysmography (Colin7000, Komaki, Japan). The Et-CO_2_ was measured by an infrared capnograph (Capnograph, Model 9004, Smith Medical, USA) using the standard protocol.

The capnograph was connected to a breathing mask and to the computer. Et-CO_2_ signals were recorded on the same time scale as other variables. This enabled us to compare the signals and perform correlations between them. The experiment lasted 40 min, consisting of a 10 min baseline period, a 20 min period during which an intravenous infusion of CGRP 1.5 mcg/min (Calbiochem, Merck4Biosciences, Darmstadt, Germany) was administered, and a 10 min period after the end of the application of CGRP.

**Figure 1:**
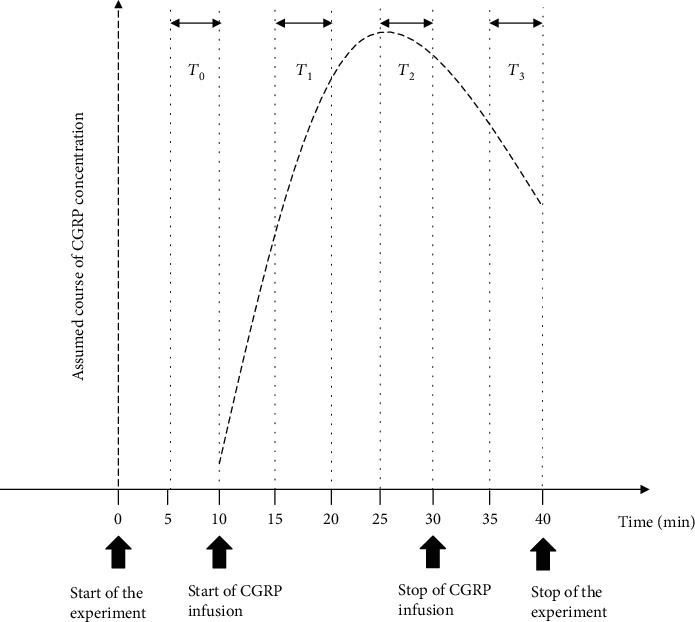
Flowchart of the experiment. CGRP: calcitonin gene-related peptide.

The TCD Multi-Dop X4 software (DWL, Sipplingen, Germany) was used to define the average values of all parameters (vm MCA, MAP, HR, and Et-CO_2_) during 5 min intervals. *T*_0_ represented the interval during the last part of the baseline period (5 to 10 min of the experiment), *T*_1_ was the 5 min interval in the first part of CGRP infusion (15 to 20 min of the experiment), *T*_2_ represented the 5 min interval in the last part of CGRP infusion (25 to 30 min of the experiment), and *T*_3_ was the 5 min interval in the last part of the experiment after the end of CGRP infusion (35 to 40 min of the experiment). The intervals *T*_0_, *T*_1_, *T*_2_, and *T*_3_ were used as measuring points. We represent the flowchart of the experiment in Figure 1.

The TCD software enabled us to calculate an average integral for each 5 min interval using the following equation for vm MCA:

(1) $$\begin{array}{c} \text{vm MCA}=\int vdt/\left(t_{0\min}–t_{5\min}\right). \end{array}$$

The mean values of other variables (MAP, HR, and Et-CO_2_) were also calculated for the same time intervals as the vm MCA, using the TCD software.

In the next step, we calculated the responses of vm MCA, Et-CO_2_, HR, and MAP as differences between measuring points. The response 1 represented the difference between points *T*_1_ and *T*_0_, response 2 between points *T*_2_ and *T*_0_, and response 3 between *T*_3_ and *T*_0_. From responses 1, 2, and 3, we formed the composed variables vm MCA_tot_, Et-CO_2tot_, MAP_tot_, and HR_tot_ based on the premise that CGRP was elevated in all three responses.

CGRP-IH was detected according to the International Classification of Headache Disorders third edition [9].

For the statistical analysis, the IBM SPSS software was used (version 21, SPSS Inc., USA). Paired *t*-test and Student's *t*-test were used to test the significance of differences between dependent and independent variables. Linear regression and logistic regression were used to test the correlations between the variables. Normality of variability distribution was tested, and all variables had values in the Shapiro-Wilk test greater than 0.05. The results of the statistic tests were statistically significant if *p* < 0.05.

## 3. Results

CGRP-IH was found in 5 subjects (25%), of whom three were female and two were male. We did not find significant difference in age between the CGRP-IH (33.8 ± 2.0 years) and non-CGRP-IH groups (36.8 ± 4.4 years) (*p* = 0.158).

**Figure 2:**
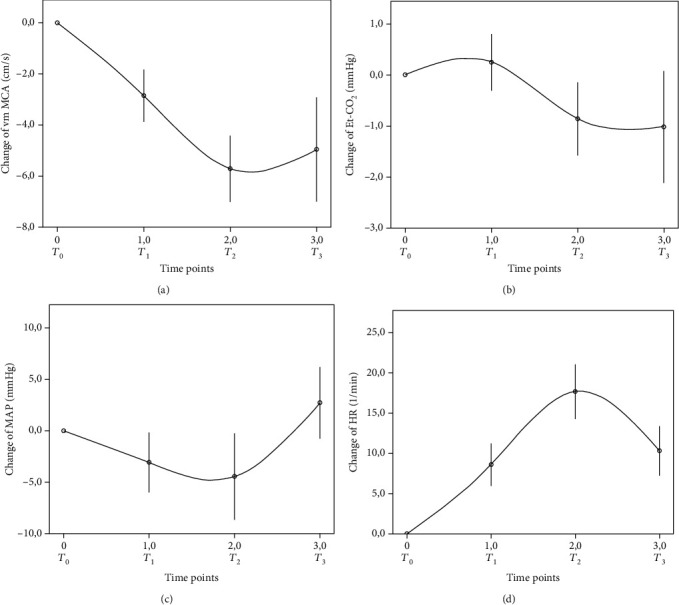
Changes in physiological variables in all participants. Et-CO_2_: end-tidal CO_2_ partial pressure; HR: heart rate; MAP: mean arterial pressure; vm MCA: mean flow velocity in the middle cerebral artery.

In Figure 2, we represent the changes in the physiological variables obtained by multimodal TCD in all participants. ANOVA for repeated measurements showed significant differences in changes of vm MCA (*p* < 0.001), Et-CO_2_ (*p* = 0.003), MAP (*p* < 0.001), and HR (*p* < 0.001).

In Table 1, the significances of differences between the measuring points are shown.

**Figure 3:**
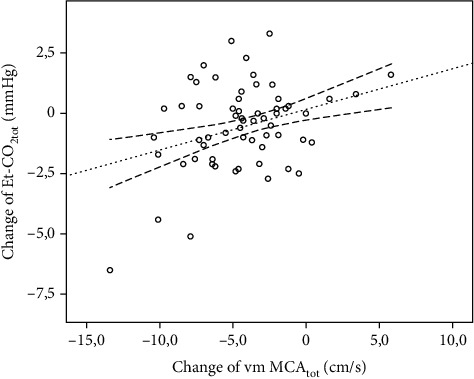
The relationship between changes of vm MCA_tot_ and Et-CO_2tot_. Et-CO_2tot_: composed end-tidal CO_2_ partial pressure; vm MCA_tot_: composed mean flow velocity in the middle cerebral artery.

We tested the relationship between the changes of vm MCA_tot_ and Et-CO_2tot_, which appears to be positive and significant (*p* = 0.005), as illustrated in Figure 3.

**Figure 4:**
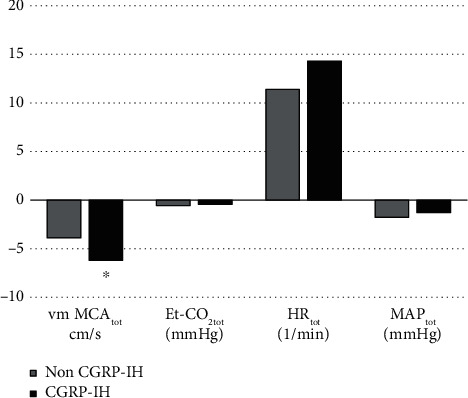
The means of changes in composed variables for non-CGRP-IH and CGRP-IH groups. ^∗^Indicates a statistically significant difference. CGRP-IH: calcitonin gene-related peptide-induced headache; Et-CO_2tot_: composed end-tidal CO_2_ partial pressure; HR_tot_: composed heart rate; MAP_tot_: composed mean arterial pressure; vm MCA_tot_: composed mean flow velocity in the middle cerebral artery.

In Figure 4, we show the means of vm MCA_tot_, Et-CO_2tot_, MAP_tot_, and HR_tot_ changes for non-CGRP-IH and CGRP-IH groups. Each column represents the sum of all time points' changes (*T*_1_, *T*_2_, and *T*_3_). A *t*-test was used to test the differences between the groups for the variables. The difference for vm MCA_tot_ changes was significant (*p* = 0.021), whereas for Et-CO_2tot_ (*p* = 0.838), MAP_tot_ (*p* = 0.839) and HR_tot_ (*p* = 0.198), they appeared to be nonsignificant.

At the end of the analysis, we tested the association between CGRP-IH and vm MCA_tot_, Et-CO_2tot_, MAP_tot_, and HR_tot_. The logistic regression showed a significant relationship between vm MCA_tot_ and CGRP-IH (OR = 0.79, 95% C.I. 0.64-0.97; *p* = 0.028), whereas Et-CO_2tot_, MAP_tot_, and HR_tot_ did not associate significantly with CGRP-IH (*p* = 0.834, *p* = 0.835, and *p* = 0.200, respectively).

## 4. Discussion

The main findings of our study are a significant relationship between vm MCA and Et-CO_2_ and a decrease in vm MCA_tot_ in subjects with CGRP-IH. Both support the vasodilatation of MCA during CGRP activity. The first finding is in the context of the regulatory role of pCO_2_ after CGRP-induced increase of CBF. Nevertheless, it does not explain the causality of the phenomena. The association could arise due to an initial decrease of Et-CO_2_ with secondary effects on CBF and consequently a decrease of vm MCA. According to the available literature, it is not known whether CGRP directly causes hypocapnia.

On the other hand, CGRP can cause CGRP-IH, which may itself trigger hyperventilation. However, CGRP-IH appeared only in a minority of the study participants. In addition, our results do not support a pronounced decrease of Et-CO_2_ in CGRP-IH. Moreover, we excluded subjects with a medication overuse habit. Furthermore, none of the participants reported a feeling of anxiety, which could be an emotional drive for hyperventilation.

Our multimodal recording provided data on the time course of Et-CO_2_ and vm MCA. The results clearly showed that the vm MCA decreased at the start of the CGRP infusion, while Et-CO_2_ decreased after the second part of the infusion. This time delay could be explained by vasodilatation of MCA causing an increase in CBF and a secondary, compensatory hyperventilatory response with a decrease in Et-CO_2_. This reasoning is supported by an objective finding in the study where it was reported that the peak decrease in Et-CO_2_ occurred 5 min after the end of the CGRP infusion [3].

The vasodilatation of MCA is supported by pharmacokinetic studies with CGRP. The pharmacokinetics of exogenous CGRP was investigated in animal and human studies [10, 11]. It was found that CGRP pharmacokinetics follows the first order with a plateau reached within one hour. The elimination of CGRP shows a two-phase, biexponential decay [12]. The half-life was found to be for the first phase, 6.9 minutes, and for the second one, 26.4 minutes, which supports a modulatory role of CGRP. Translation of basic studies to the findings explains why the response of vm MCA increased during the CGRP infusion and decreased after it.

The second evidence for MCA dilatation is a significant decrease of vm MCA in CGRP-IH participants compared to non-CGRP-IH participants. This finding is in accordance with the prediction based on TVS. It is widely accepted that the CGRP molecule is associated with TVS [13]. Generally, migraine headaches are linked to TVS and neurogenic inflammation [14]. The systemic administration of CGRP to migraine sufferers triggers a migraine-like attack phenotypically similar to the subject's spontaneous attack [2]. On the other hand, a CGRP infusion to nonmigraineurs does not provoke migraine-like attacks [15]. In the study, we did not observe typical migraine-like attacks with prominent photo- and phonophobia, but in some cases, an immediate widespread headache emerged followed by nausea. Therefore, some degree of sensitization to neurogenic inflammation could occur even in nonmigraineurs. Furthermore, sensitization may precede in a latent form, and when the pain threshold is reached, headaches and migraines are evoked. Thus, it is possible that in some nonmigraineurs, the pain threshold is lower or the baseline sensitization is higher [4]. In those with a lower threshold, exogenically introduced CGRP might evoke CGRP-IH.

Although the vasodilatory properties of CGRP are well documented, its somatosensory function regarding the modulation of neuronal sensitization and of enhanced pain has received considerable attention recently. The observations suggest that meningeal arteries can be dilated by CGRP when this compound is administered locally or released by activation of the trigeminal nerve [16]. The neurogenic inflammation can be achieved by mast cell degranulation. It is doubtful whether CGRP can induce degranulation of meningeal mast cells (MCs). Some studies have reported that CGRP had no direct modulatory effects on meningeal MC activation. Ongoing activation of meningeal MCs is not mediated by peripheral CGRP signaling and does not contribute to the development of evoked cephalic mechanical pain hypersensitivity in a rat model [17]. Studies in rodents suggest that the potential involvement of CGRP and the ensuing activation of meningeal MCs and resident immune cells can activate the headache pain pathway [18]. Thus, the question whether exogenous CGRP can induce neurogenic inflammation as part of TVS remains open.

We followed changes in systemic circulation monitoring HR and MAP during and after CGRP infusion. We observed a significant decrease in MAP during the infusion and normalization after the end of it. As expected, HR increased during CGRP infusion and decreased after the end of it. According to previous studies, intravenous administration of CGRP leads to positive chronotropic and inotropic effects associated with a reduction in blood pressure and the rise of plasma noradrenalin and adrenalin levels [19]. The subsequent sympathetic activation with a release of catecholamines displays the indirect effects of CGRP, which we clearly observed in healthy subjects. In general, the level of CGRP in the systemic circulation in humans is limited to the picomolar range [20] and in this range, CGRP is not thought to have a systemic effect on the vascular tissue. However, as mentioned, the plasma concentration of CGRP is elevated during migraine attacks associated with neurogenic inflammation in the brain [21], draining CGRP from the brain to the systemic circulation due to trigeminovascular sensitization. Therefore, our systemic findings are as expected and are in accordance with the existing knowledge.

The limitations of the study are principally the small number of participants and the lack of data on CGRP plasma concentration, which, if available, could clarify our thesis on latent sensitization in nonmigraine subjects. Using the nocebo arm in our study could further improve the reliability of our assumptions.

## 5. Conclusions

Our study provides additional evidence for vasodilatation of MCA due to intravascular CGRP detected by multimodal TCD. In the context of TVS, MCA vasodilation seems to represent an epiphenomenon of the underlying TVS. While the pain threshold depends on the cognitive features of the subjects, CGRP provocation with TCD polymodal monitoring enables us to discriminate between the psychologic and biologic effects on headache occurrence. In addition, by using the human experimental headache model, previously performed studies have shown that headache response and MCA changes make up a valid model for investigation of migraine pathogenesis.

## Figures and Tables

**Table 1:** Significance of differences between the measuring points.

Parameter	*T*_0_-*T*_1_	*T*_0_-*T*_2_	*T*_0_-*T*_3_
vm MCA (cm/s)	*p* < 0.001	*p* < 0.001	*p* < 0.001
Et-CO_2_ (mmHg)	*p* = 0.376	*p* = 0.023	*p* = 0.066
MAP (mmHg)	*p* = 0.062	*p* = 0.027	*p* = 0.119
HR (1/min)	*p* < 0.001	*p* < 0.001	*p* < 0.001

Et-CO_2_: end-tidal CO_2_ partial pressure; HR: heart rate; MAP: mean arterial pressure; vm MCA: mean flow velocity in the middle cerebral artery.

## Data Availability

The data used to support the findings of this study are available from the corresponding author upon request.
